# Sonographic and Endoscopic Findings in Cocaine-Induced Ischemic Colitis

**DOI:** 10.1155/2015/680937

**Published:** 2015-12-21

**Authors:** Thomas Leth, Rune Wilkens, Ole K. Bonderup

**Affiliations:** ^1^Section of Gastroenterology and Section of Radiology, Diagnostic Centre, University Research Clinic for Innovative Patient Pathways, Silkeborg Regional Hospital, 8600 Silkeborg, Denmark; ^2^Department of Hepatology and Gastroenterology, Aarhus University Hospital, 9000 Aarhus C, Denmark

## Abstract

Cocaine-induced ischemic colitis is a recognized entity. The diagnosis is based on clinical and endoscopic findings. However, diagnostic imaging is helpful in the evaluation of abdominal symptoms and prior studies have suggested specific sonographic findings in ischemic colitis. We report sonographic and endoscopic images along with abdominal computed tomography in a case of cocaine-induced ischemic colitis.

## 1. Introduction

Ischemic colitis predominantly occurs in elderly patients with atherosclerosis or diabetes [1]. Cocaine is a potent vasoconstrictor and cocaine-induced ischemic colitis has previously been reported in the literature [2]. The condition must be considered in patients with abdominal pain and current cocaine abuse. Abdominal CT-scan is typically the choice of diagnostic imaging in patients with acute, abdominal pain; however, ultrasound plays an increasing role in this situation [3]. Attention to any pathological change in the intestine at ultrasound is essential. We describe a case of cocaine-induced ischemic colitis where the diagnosis was suggested based on an abdominal ultrasound.

## 2. Medical Case History

**Figure 1:**
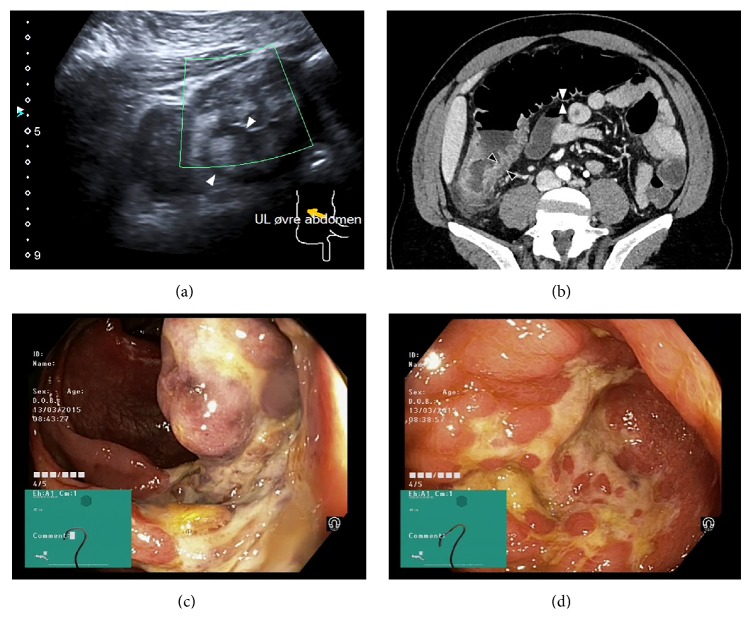
(a) Transabdominal ultrasound. Cecal lumen is compromised by the thickened bowel wall. (b) Computed tomography scan of the right colon. Ascending colon is thickened with intramural edema. (c) Endoscopic image from ascending colon. Image shows ischemic transformation with edema and ulcerations. (d) Endoscopic image from cecum. Circumferential alterations with fibrin-covered ulcerations.

A 41-year-old man with a history of drug abuse was admitted with diffuse abdominal pain and bloody stools. One year previously the patient was diagnosed with hepatitis C, genotype 1A with a positive HCV RNA. A fibroscan had revealed normal values without indications of cirrhosis. Owing to lack of compliance no treatment of HCV was initiated. On admission physical examination revealed tenderness in the right lower quadrant with a maximum at McBurney's point. The patient was feverish with a body temperature of 38.3°C and had elevated C-reactive protein at 14 mg/dL and a white blood cell count of 14,500/dL. Appendicitis was suspected and the patient was referred to an abdominal ultrasound, which revealed a typical case of isolated right sided ischemic colitis (Figure 1(a)). Due to the young age of the patient he was questioned about ongoing drug abuse linking cocaine abuse to ischemic colitis. Detailed history revealed a drug abuse including the use of intravenous cocaine several times weekly during the last three months. The patient described intermittent and self-limiting abdominal pain and vomiting during the same period. The most recent cocaine administration was the day prior to admission. At the day of admission, the symptoms were more pronounced leading to hospitalization.

Based on the clinical assessment and diagnostic imaging appendicitis could be ruled out. A subsequent contrast enhanced abdominal computed tomography showed an inflamed ascending colon and cecum possibly extending into the terminal ileum (Figure 1(b)).

To confirm the diagnosis and to rule out Crohn's disease the patient underwent a colonoscopy. The ascending colon (Figure 1(c)) and cecum (Figure 1(d)) showed ulcerations whereas the remaining colon appeared normal. Histology on the biopsies revealed changes consistent with ischemic colitis without signs of inflammatory bowel disease or malignancy.

Six days after the debut of symptoms a follow-up abdominal ultrasound scan could no longer prove any bowel wall thickening. The patient was treated with analgesics and experienced a gradual recovery towards discharge 11 days after admission. A MR enterography performed 13 days after admission confirmed normalization of the bowel. The patient was lost to follow-up at the next appointment.

## 3. Discussion

Bowel ischemia on an atherosclerotic background is most often seen in elderly patients. If there are signs of bowel ischemia in younger patients without predisposing conditions then other possible causes including cocaine abuse must be considered. Cocaine-induced ischemic colitis is previously reported and this case report underlines the importance of obtaining a thorough and detailed drug abuse history from patients presenting with abdominal pain. Early diagnosis is crucial due to the high morbidity and mortality of the condition [4]. The condition is usually treated conservatively, but, in the case of a complicating perforation or peritonitis, a surgical procedure may be required.

This case report illustrates that early suspicion of cocaine-induced bowel ischemia is important for ruling out differential diagnoses like appendicitis or inflammatory bowel disease. Based on a careful medical history and diagnostic imaging unnecessary and acute surgical procedures were avoided.

Ultrasonography is a noninvasive examination increasingly used for the initial assessment of patients with acute abdominal symptoms. At ultrasound, the typical signs of ischemic colitis are a substantial circumferential bowel wall thickening with a segmental distribution of 10–30 centimeters. Often layering of the bowel walls is preserved and no or only weak color Doppler imaging signals are seen. Fibrofatty proliferation of the mesentery, usually seen as abundant hyperechoic fat surrounding an inflamed bowel (e.g., diverticulitis or creeping fat in Crohn's disease), is limited [5].

The pathophysiological course of ischemic colitis includes increased mucosal permeability, local edema, and inflammation with subsequent ulcerating disease after reestablishment of perfusion resulting in further increase in wall thickness and inflammation [1, 6]. The typical localization of thromboembolic bowel ischemia is in the left side of the colon. On the contrary, cocaine-induced vasoconstriction causing bowel ischemia is more frequently localized in the cecum and ascending colon [1, 5] due to a general less developed and smaller vasa recta, more prone to vasoconstriction than in the left colon [5]. Further isolated right sided colon ischemia has shown an overall worsened outcome compared to ischemic colitis elsewhere [7]. In patients with infectious colitis diagnostic imaging shows more diffuse changes with signs of increased blood flow. Bowel wall thickening of more than 10 mm without concurrent substantial mesenteric proliferation and increased blood flow in areas with active inflammation is rarely seen in Crohn's disease.
